# Using a Process Dissociation Approach to Assess Verbal Short-Term Memory for Item and Order Information in a Sample of Individuals with a Self-Reported Diagnosis of Dyslexia

**DOI:** 10.3389/fpsyg.2016.00208

**Published:** 2016-02-24

**Authors:** Xiaoli Wang, Yifu Xuan, Christopher Jarrold

**Affiliations:** ^1^School of Psychology, Northwest Normal UniversityLanzhou, China; ^2^School of Experimental Psychology, University of BristolBristol, UK

**Keywords:** dyslexia, item memory, order memory, short-term memory, process dissociation

## Abstract

Previous studies have examined whether difficulties in short-term memory for verbal information, that might be associated with dyslexia, are driven by problems in retaining either information about to-be-remembered items or the order in which these items were presented. However, such studies have not used process-pure measures of short-term memory for item or order information. In this work we adapt a process dissociation procedure to properly distinguish the contributions of item and order processes to verbal short-term memory in a group of 28 adults with a self-reported diagnosis of dyslexia and a comparison sample of 29 adults without a dyslexia diagnosis. In contrast to previous work that has suggested that individuals with dyslexia experience item deficits resulting from inefficient phonological representation and language-independent order memory deficits, the results showed no evidence of specific problems in short-term retention of either item or order information among the individuals with a self-reported diagnosis of dyslexia, despite this group showing expected difficulties on separate measures of word and non-word reading. However, there was some suggestive evidence of a link between order memory for verbal material and individual differences in non-word reading, consistent with other claims for a role of order memory in phonologically mediated reading. The data from the current study therefore provide empirical evidence to question the extent to which item and order short-term memory are necessarily impaired in dyslexia.

## Introduction

Developmental dyslexia has been defined as a specific difficulty in learning to read that cannot be attributed to general intellectual difficulties, sensory disorders, or poor schooling (Critchley, 1975; Ramus, 2014). Developmental dyslexia is often accompanied by a deficit in verbal short-term memory. Indeed, the association of dyslexia and verbal short-term memory deficits has been identified in both children and adults by a large number of researchers (e.g., Brady et al., 1983; Pennington et al., 1990; Avons and Hanna, 1995; Snowling et al., 1996; Kramer et al., 2000; Roodenrys and Stokes, 2001; Plaza et al., 2002; Ramus et al., 2003; Tijms, 2004; Nithart et al., 2009; Martin et al., 2010).

However, the nature of this association is still not fully understood. One potential reason for this is that, though simple, typical verbal short-term memory tasks may measure more than one ability. In particular, in a typical verbal short-term memory span task, two types of information have to be remembered –item information and the serial order in which the items are presented.

A number of studies have suggested that item memory and order memory are two distinct processes (cf. Healy, 1974). For example, both phonological similarity and semantic similarity produce dissociative effects on item and order memory (e.g., Murdock, 1976; Crowder, 1979; Saint-Aubin and Poirier, 1999; Nairne and Kelley, 2004). Majerus et al. (2006) found that short-term memory tasks maximizing order or item recall were in dependently associated with vocabulary acquisition in children. Some researchers have argued that order memory might play a more important role than item memory in the acquisition of vocabulary and reading because it ensures the ordered reactivation of phonological representation in the language network, which in turn increases the probability that the phonological representation of a new word is transformed into a stable representation in long-term memory (Baddeley et al., 1998; Gupta, 2003). The neuropsychological literature provides more evidence for the distinction of item and order processes in short-term memory. Selective impairments of either item or order memory capacities have been observed in children with Down syndrome (Brock and Jarrold, 2004; though see also Smith and Jarrold, 2014), patients with semantic dementia (Majerus et al., 2007), and patients with aphasia (Attout et al., 2012).

Therefore, the distinction between item and order memory is critical for understanding verbal short-term memory deficits in dyslexia. It has been assumed that most individuals with dyslexia suffer from inefficient phonological representations (Snowling, 1981; Ramus et al., 2003), resulting in poor performance in verbal short-term memory tasks. From the perspective of separable order and item memory processes, the suggestion that verbal short-term memory deficits in dyslexia are a consequence of impaired phonological representations implies an item memory deficit, because item information depends directly on the quality of the language network (Burgess and Hitch, 1999; Majerus and D’Argembeau, 2011).

However, although verbal short-term memory deficits in dyslexia have been mostly explored using tasks in which both item and serial order recall are involved (e.g., Snowling et al., 1996; Kramer et al., 2000; Tijms, 2004), there is also evidence to imply an association between order memory deficits and reading impairment (Mason, 1980; Brady et al., 1983; Nithart et al., 2009). More recently, studies have attempted to examine item and order processes separately. Martinez Perez et al. (2012) found that children with dyslexia performed significantly less well than a chronological age matched control group on an item memory measure, but less well than *both* chronological age and reading age matched control groups on a serial order short-term memory task. Furthermore, Martinez Perez et al. (2013) observed impaired short-term memory for order information in adults with dyslexia that was independent of any short-term memory impairment for item information. Bogaerts et al. (2015) found that dyslexic participants showed both a long-term serial order learning difficulty and impaired short-term memory for order information. They also observed reliable lexicalization of a repeated sequence of phoneme combinations among their control group but not among the dyslexic group, suggesting that a longer-term serial-order learning impairment may lead to impaired lexical representations. They proposed that the evidence in support of a phonological impairment in dyslexia might, at least partly, be explained in terms of problematic serial-order representation and learning (Szmalec et al., 2011). Thus, these studies suggest a severe impairment of short-term memory for order information that cannot be reduced to a phonological representation deficit in both children and adults with dyslexia.

However, this conclusion needs to be qualified for both methodological and theoretical reasons. First, pure measurements of item and order memory are required to properly investigate the nature of any association between these short-term memory skills and dyslexia. However, most short-term memory tasks tap both processes in combination. Researchers have attempted to maximize either the item or order memory processes required in the tasks employed in their studies, but it is still far from clear that these represent pure measurements of item or order memory (cf. Nairne and Kelley, 2004). Another issue is whether short-term memory for order information is entirely language-independent. There remains dispute as to whether order memory rests on domain-general or domain-specific process. Some have argued that order memory is language-independent (Henson, 1998; Brown et al., 1999; Burgess and Hitch, 1999; Gupta, 2003). However, it has been shown that some language characteristics of to-be-remembered word lists, such as phonological and semantic similarity, affect individuals’ order memory. For example, both phonological similarity and semantic similarity affect order memory (e.g., Murdock, 1976; Crowder, 1979; Nairne and Kelley, 2004), suggesting that order memory process might not be entirely language-independent.

Furthermore, it has been suggested that words (particularly irregular words) and non-words are read via two types of cognitive pathways, one involving semantic representations and the other connecting orthography with phonology directly. Whether reading really involves two entirely distinguishable routes is a subject of debate, and the links between orthographic, phonological, and semantic codes have been successfully instantiated in connectionist models of reading (Plaut et al., 1996; Harm and Seidenberg, 2001; Harm and Seidenberg, 2004). More recently, Tree and Kay (2006) provided evidence from a single case of an individual with acquired dyslexia who did not show generalized phonological processing impairments. They therefore suggested that sub-lexical problems in reading that compromise a direct link between orthography and phonology (cf. Coltheart et al., 2001) can exist separately of more general linguistic difficulties. In sum, there are a number of possible pathways, that depend more on either phonological, semantic, or language-independent processes, that might be involved in reading, and which might be differently associated with item and order memory deficits in individuals with dyslexia.

The present study therefore aimed to explore short-term memory for both item and order information in adults with a previous diagnosis of dyslexia. A process dissociation paradigm was employed in order to obtain purer estimates of these abilities than those derived from any previous work and to properly assess whether individuals with a previous diagnosis of dyslexia experience an item or an order short-term memory impairment, or both. In addition, language characteristics, specifically semantic similarity and phonological similarity, were manipulated to explore the interactions between item and order memory and these language features. If dyslexia is characterized by a general and independent verbal short-term memory deficit, that is not simply a consequence of poor phonological processing abilities, then both item and order memory deficits would be expected in adult participants with a previous diagnosis of dyslexia. Furthermore, if any order memory deficit is language-independent, then one would expect order memory estimates among individuals who had received a diagnosis of dyslexia to be no more affected by ‘language-related’ manipulations of phonological or semantic similarity than is seen in typical readers.

The process dissociation paradigm (Jacoby et al., 1997) was first used to obtain relatively pure measurements of item and order retention in short-term memory by Nairne and Kelley (2004). This approach assumes that two processes, in this case item and order memory, operate independently. The estimates of the two processes can be obtained by comparing performance across two experimental conditions, called inclusion and exclusion conditions respectively. In an inclusion condition both item and order processes promote performance, while in an exclusion condition one process facilitates and the other reduces performance. In Nairne and Kelley’s (2004) original study, the inclusion task was a serial recall task, in which participants were required to recall all items in their position in a sequence. As a result, this task relied on both item and order memory. Assuming independence of these two processes, the probability of a correct response is equal to the product of the probability of remembering the item (I) and the probability of remembering its position (Or). In the exclusion condition, participants were presented a sequence of items, and then required to recall all of them with the exception of the one that appeared in a particular position. In this case, participants would only recall the item from this position if they remembered the item but forgot its order. The following two formulas can be obtained from the two conditions:

Inclusion = I^∗^Or

Exclusion = I^∗^(1-Or)

The estimates of item memory (I) and order memory (Or) can then be obtained by simple algebra.

I = inclusion + exclusion

Or = inclusion/I.

As noted above, the process dissociation paradigm depends on the assumption that the two processes that are set in opposition in the exclusion task are independent of one another. A number of computational models of verbal short-term memory employ separate processes for the representation of item and order information (e.g., Burgess and Hitch, 1992; Brown et al., 2000), and the assumption that item and order memory are entirely independent of each other is central to the perturbation model of short-term memory (Estes, 1997). In Nairne and Kelley’s (2004) study, simulations using the perturbation model provided a very good fit to the data, providing some support for the validity of this assumption of independence. However, it should be noted that not all would agree with the claim that item and order information are entirely separable (e.g., Farrell and Lewandowsky, 2002).

Since relatively short lists were used in Nairne and Kelley’s (2004) inclusion and exclusion tasks, participants had to complete simple addition problems after presentation of the memoranda to avoid ceiling effects on serial recall in that study. To remove this problem, and to use tasks that were more similar to standard short-term memory measures without a filled delay, adapted tasks were used in present study. Specifically, rather than delaying recall by the inclusion of addition problems between presentation and recall, immediate recall was required for longer lists of items than used by Nairne and Kelley (2004).

To properly understand the profile of item and order memory seen in adults with a previous diagnosis of dyslexia, a control group of typical readers was also included in this study. In addition to the item and order memory tests, we also administered a reading a test to all participants in order to confirm that the individuals with an existing diagnosis of dyslexia taking part in this study had expected difficulties in non-word reading relative to this control group.

## Materiels and Methods

### Participants

Fifty-eight undergraduates from University of Bristol participated in this study for course credit, or for 5 pounds remuneration. All subjects gave written informed consent, and the study was approved by the Faculty of Science Human Research Ethics Committee of the University of Bristol. All participants were native English speakers with normal vision or corrected normal vision. Twenty-eight of them were individuals with a self-reported diagnosis of developmental dyslexia, and 13 were typical readers who reported no history of reading difficulties. Individuals in the developmental dyslexia group were recruited on the basis of self-identifying with a diagnosis of dyslexia, having been recruited through advertisements placed at the University of Bristol’s Disabilities Service. All participants in this group signed a consent form to confirm that they had received a formal diagnosis of dyslexia from a qualified practitioner, and approximately half the sample spontaneously brought evidence of this diagnosis to the testing session. However, given that a formal diagnosis could not be fully confirmed in all cases, reading ability was explicitly checked by administering a reading test as noted above. One of the typical group showed very poor performance on the reading test, and so the analysis below excluded this participant.

### Materials

#### Reading Ability Test

Fifteen regular words (words with regular pronunciation rules), 15 irregular words (words without regular pronunciation rules), and 15 non-words developed by Manis et al. (1996) were selected for the reading ability test. Three blocks were constructed and each block consisted of 15 words of the same type and the sequence of blocks was counterbalanced between participants. Participants were required to read aloud the items as accurately as possible.

#### Experimental Tasks

Phonological materials: 108 nouns were used to construct 18 word sets; each set were constructed with MRC Psycholinguistic Database and consisted of six phonologically similar rhyming nouns. Eighteen dissimilar sets were constructed by simply combining words from the similar sets in the following way: the 18 similar lists were divided into three groups of six sets of six words; these groups were named as G1, G2, and G3. Within each group, six words, one from each set, were selected and combined to create a list of phonologically dissimilar items. Thus, 18 phonologically similar lists and 18 dissimilar lists were obtained and used in the inclusion condition. The order of similar and dissimilar trials was randomly determined in the task. The same word sets were used in the exclusion condition with the words in a list and sequence of trials re-randomized. Kucera–Francis written frequency scores were available for all 108 words, and the set had a mean frequency score of 80.06 (*SD* = 136.09). The word printed familiarity ratings derived from MRC Psycholinguistic Database (Coltheart, 1981) were available for 95 of these words (mean = 543.42, *SD* = 53.43).

Semantic materials: 108 nouns selected from 18 semantic categories (e.g., vegetables, countries, drinks) were used to construct similar and dissimilar lists for semantic versions of the inclusion and exclusion tasks. The categories were constructed with reference to the UMBC Semantic Similarity Service developed by Han et al. (2013). The semantically dissimilar lists were constructed in the same way as the phonologically dissimilar lists. Latent Similarity Analysis (LSA) (Landauer et al., 1998) similarity ratings were used to compare the semantic similarity between the resultant similar and dissimilar sets. This showed that the mean LSA similarities of semantic similar lists (0.326) was significant greater than that of semantic dissimilar lists (0.086), *F*(1,34) = 44.376, *p* < 0.001, *MSE* = 0.012, η^2^ = 0.566. Kucera–Francis frequency values were available for 100 of these 108 words, and this subset had a mean frequency score of 62.60. The word printed familiarity ratings derived from MRC Psycholinguistic Database (Coltheart, 1981) were available for 78 of the words (mean = 571.13, *SD* = 37.44).

### Design and Procedure

This experiment employed a within-subjects design, with both groups being administered both the reading ability test and experimental tasks.

In the reading ability test, each item (regular word, irregular word, or non-word) was presented as a written word in the middle of the 14-inch screen on a laptop in Arial font size 44 in black on a white background. The participants were required to read aloud the item on screen, and then the next item appeared on the screen after the experimenter advanced the computer program. The proportions of correct responses for each type of word were recorded as the reading scores.

For the experimental task, all participants contributed data to all cells of the design manipulation type (semantic vs. phonological) × similarity (similar vs. dissimilar) × condition (inclusion vs. exclusion). The experimental task consisted of four blocks, phonological inclusion, phonological exclusion, semantic inclusion and semantic exclusion condition respectively. Each block consisted of 36 trials containing18 similar lists and 18 dissimilar lists. The sequences of blocks were counterbalanced between-subjects.

An adapted serial recall task was employed as the inclusion task. In a trial, a list of six words was presented in order on the screen. Each item was presented visually as a written word in Calibri font size 44 in the middle of the screen for 750 ms with a 250 ms interval between items. After presentation of a list, the response screen was shown: six blue squares appeared in a horizontal line in the middle of the screen. Participants were required to recall all words in their presented order by verbal report. When they recalled the words they were required to touch one of the squares on the screen to signify the corresponding serial position or had to say “pass” for any forgotten item.

In the exclusion condition, the presentation of memoranda was exactly the same as in the inclusion condition. The response screen was similar to that used in the inclusion condition with the exception that an “X” was shown in one of the six squares. The participants were instructed to recall all the other words except for the position “X”. These remaining items could be recalled in any order. The to-be-excluded item was sampled equally often from each of the six serial positions across trials.

## Results

### Reading Test

**Table 1** summarizes the performance of the self-diagnosed dyslexia and control groups on the three blocks of the reading test. A mixed analysis of variance, with word type as the within-participants factor and group as the between-participants factor, was conducted on the reading accuracy data. It revealed a significant main effect of word type, *F*(2,110) = 87.713, *p* < 0.001, *MSE* = 2.828, η^2^ = 0.527, a significant main effect of group, *F*(1,55) = 10.927, *p* = 0.002, *MSE* = 0.006, η^2^ = 0.017, and a significant interaction between word type and group, *F*(2,110) = 7.505, *p* = 0.001, *MSE* = 2.828, η^2^ = 0.045. Within subject contrasts showed that regular word reading accuracy was significantly greater than that for irregular words and non-words (*p* < 0.001), while irregular word and non-word reading were of a comparable difficulty (*p* = 0.800). *Post hoc* tests revealed that the individuals with a self-referred diagnosis of dyslexia showed impaired irregular word reading relative to the control group, *F*(1,55) = 4.296, *p* = 0.043, *MSE* = 3.676, η^2^ = 0.072, and an even more marked deficit on non-word reading, *F*(1,55) = 12.481, *p* = 0.001, *MSE* = 5.740, η^2^ = 0.185. Levene’s test of homogeneity of variances showed that group variances were not significantly different in either of these analyses; irregular word reading, *F*(1,55) = 0.298, *p* = 0.587, non-word reading *F*(1,55) = 3.583, *p* = 0.064. Reading accuracy for regular words did not differ significantly between the two groups, *F*(1,55) = 1.374, *p* = 0.246, *MSE* = 0.408, η^2^ = 0.024, though in this case Levene’s test of homogeneity of variances was significant, *F*(1,55) = 9.410, *p* = 0.003.

**Table 1:** Background reading skill in the self-diagnosed dyslexia and control groups; proportion correct scores.

Variable	Self-diagnosed dyslexia		Control	
	Mean	*SD*	Mean	*SD*
Regular word reading	0.983	0.029	0.970	0.052
Irregular word reading	0.698	0.139	0.768	0.116
Non-word reading	0.664	0.185	0.814	0.130


### Initial Analysis of Data from the Experimental Task

Prior to calculating item and order memory estimates by the process dissociation method, an initial analysis was conducted to examine whether the data pattern observed in this study was similar to that seen in Nairne and Kelley’s (2004) results.

**FIGURE 1:**
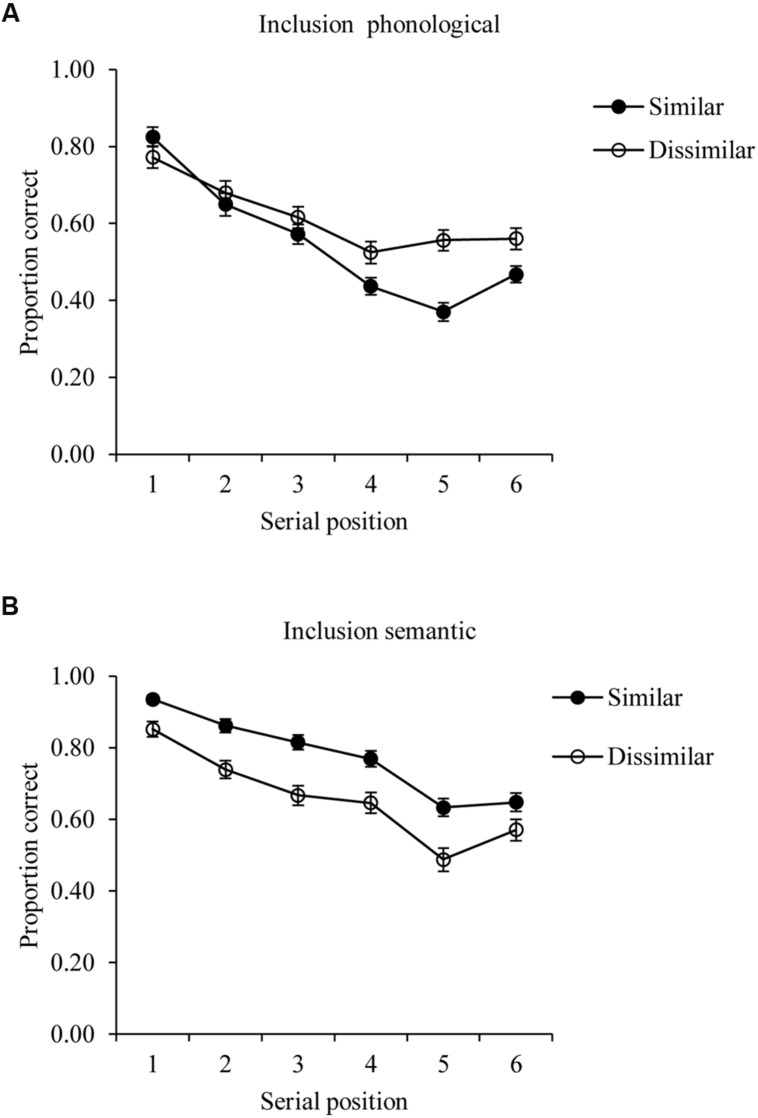
**Inclusion task performance on phonological (A) and semantic (B) conditions**. Data averaged across all participants.

**Figure 1** plots mean levels of recall for phonologically or semantically similar and dissimilar trials in the inclusion condition averaged across all participants in both groups.

For the inclusion condition, the data from the phonologically similar and dissimilar trials were subjected to a repeated measurement analysis of variance with position (1–6) and similarity as within-subject factors. This revealed a significant phonological similarity main effect, *F*(1,56) = 19.859, *p* < 0.001, *MSE* = 0.034, η^2^ = 0.017, and a significant serial position main effect, *F*(5,280) = 37.970, *p* < 0.001, *MSE* = 0.050, η^2^ = 0.244, as well as a significant interaction between serial position and similarity, *F*(5,280) = 17.328, *p* < 0.001, *MSE* = 0.010, η^2^ = 0.023. **Figure 1** shows bow-shaped serial position curves and a trend of an increasing phonological similarity effect with increasing serial position. Comparable patterns were found in Nairne and Kelley’s (2004) study using a filled delay before recall, and also are typically found in immediate serial recall, suggesting that our immediate recall and increased list length did not change the effects of similarity in fundamental way compared with Nairne and Kelley’s (2004) paradigm.

An overall analysis of variance with position and semantic similarity as within-subject factors on the data from the semantic trials showed a significant similarity main effect, *F*(1,56) = 124.997, *p* < 0.001, *MSE* = 0.018, η^2^ = 0.065, and a significant serial position main effect, *F*(5,280) = 51.461, *p* < 0.001, *MSE* = 0.032, η^2^ = 0.238. **Figure 1** shows generally bowed-shaped serial position curves, in line with Nairne and Kelley’s results. As expected, a beneficial effect for semantic similarity was seen, as has been observed in previous immediate serial recall data (see Saint-Aubin and Poirier, 1999). The interaction of semantic similarity by serial position was significant, *F*(5,280) = 2.707, *p* = 0.021, *MSE* = 0.010, η^2^ = 0.004, although **Figure 1** does not show any obvious trend in the mean data aside from a potential reduction of the effect at the final serial position.

**FIGURE 2:**
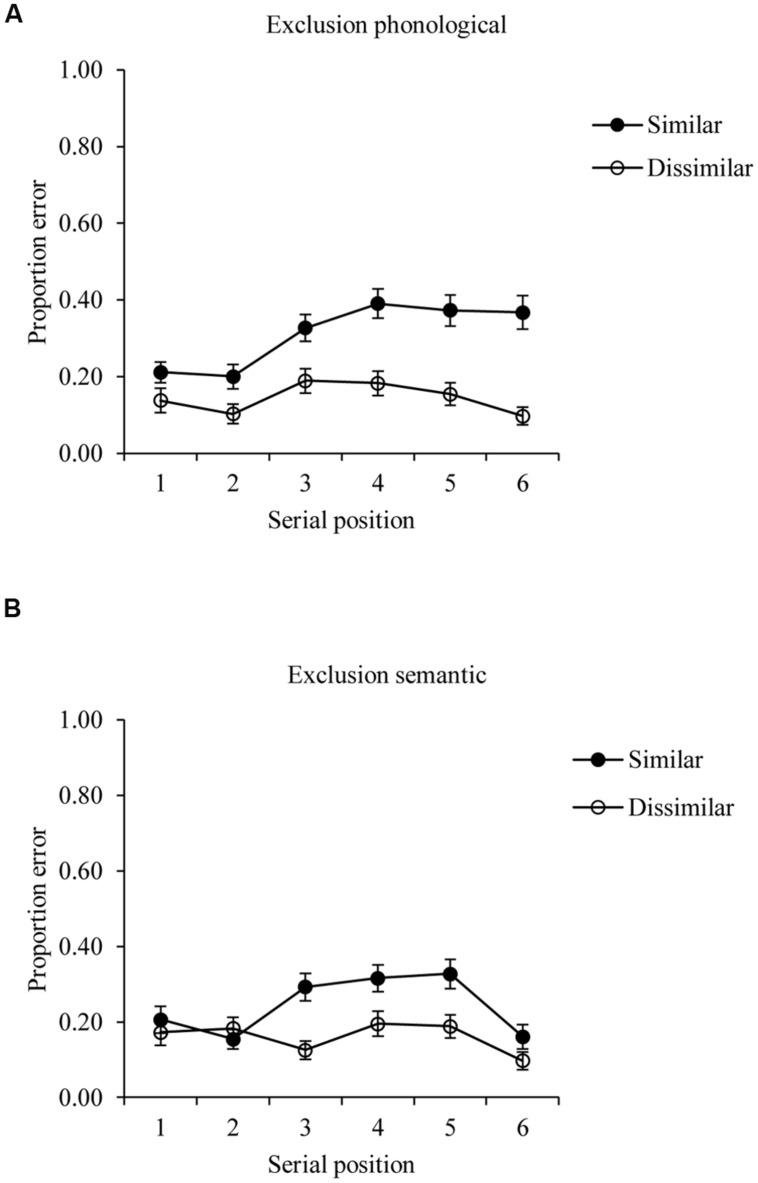
**Exclusion task performance on phonological (A) and semantic (B) conditions**. Data averaged across all participants.

The error rates from the exclusion condition are shown in **Figure 2**. As with the analysis of data from the inclusion condition, a repeated measurement analysis of variance was administered to examine the effects of phonological and semantic similarity, of serial position, and the interaction between these factors. Since it was assumed that an error in exclusion condition reflects the failure of order memory, more exclusion errors for phonological similar lists were expected (Nairne and Kelley, 2004). The analysis of data from the exclusion trials with phonological materials showed a significant main effect of phonological similarity as expected, *F*(1,56) = 65.685, *p* < 0.001, *MSE* = 0.075, η^2^ = 0.098, a significant serial position effect, *F*(5,280) = 5.567, *p* < 0.001, *MSE* = 0.062, η^2^ = 0.034, and a significant interaction, *F*(5,280) = 3.818, *p* = 0.002, *MSE* = 0.047, η^2^ = 0.018. In contrast to Nairne and Kelley’s (2004) study, in which fewer exclusion errors occurred in the first and last items in both conditions, the current serial position curves showed fewer errors for the first two and last items in the phonologically similar lists only. This might reflect the change in procedure used in the current study, with immediate rather than delayed recall leading to fewer floor effects in the easier, dissimilar, condition.

The ANOVA on data from semantic trials revealed a significant similarity main effect, *F*(1,56) = 19.896, *p* < 0.001, *MSE* = 0.055, η^2^ = 0.026, with more errors on semantically similar lists, a significant serial position effect, *F*(5,280) = 5.579, *p* < 0.001, *MSE* = 0.052, η^2^ = 0.034, and a significant interaction between semantic similarity and serial position, *F*(5,280) = 2.794, *p* = 0.018, *MSE* = 0.050, η^2^ = 0.017. The interaction was driven by less of a similarity effect at the first two and the last serial positions, consistent with Nairne and Kelley (2004).

In summary, the overall patterns of performance found in both the self-diagnosed dyslexia and the control group in this study were quite similar to those obtained by Nairne and Kelley (2004) and to the predicted patterns that emerged from their simulation of their data using the Perturbation Model (Lee and Estes, 1981; Estes, 1997). Therefore, despite the changes made to the current design in comparison to this earlier study, it is reasonable to assume that the current data will provide equally meaningful estimates of item and order memory.

### Item and Order Estimates Derived from the Process Dissociation Procedure

**FIGURE 3:**
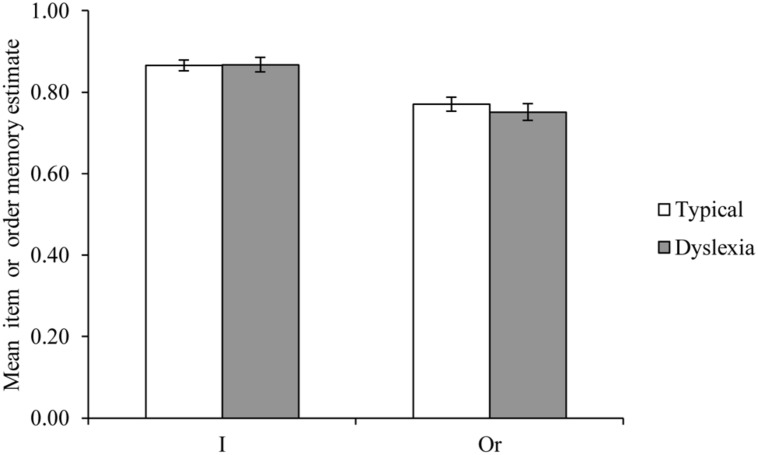
**Item memory and Order memory estimates for the self-diagnosed dyslexia and typical groups**.

Item and order memory estimates were calculated for each participant from their average performance across materials and similarity conditions using the equations shown above. The resultant average item and order estimates for both groups are shown in **Figure 3**. Analysis of variance of these data revealed that both item and order estimates were not significantly different across the two groups; for item memory, *F*(1,55) = 0.006, *p* = 0.940, *MSE* = 0.010, η^2^ < 0.001, and for order memory, *F*(1,55) = 0.712, *p* = 0.402, *MSE* = 0.007, η^2^ = 0.013. In order to better understand these null effects, a Bayesian analysis was conducted following the procedures outlined by Masson (2011). This showed that the posterior probability in favor of the null hypothesis was 0.883 for the comparison of item memory estimates and 0.842 for the comparison of order memory estimates. According to Raftery (1995) this amounts to ‘positive’ (as opposed to either ‘weak’ or ‘strong’) evidence for the null hypothesis of no group difference in each case.

**FIGURE 4:**
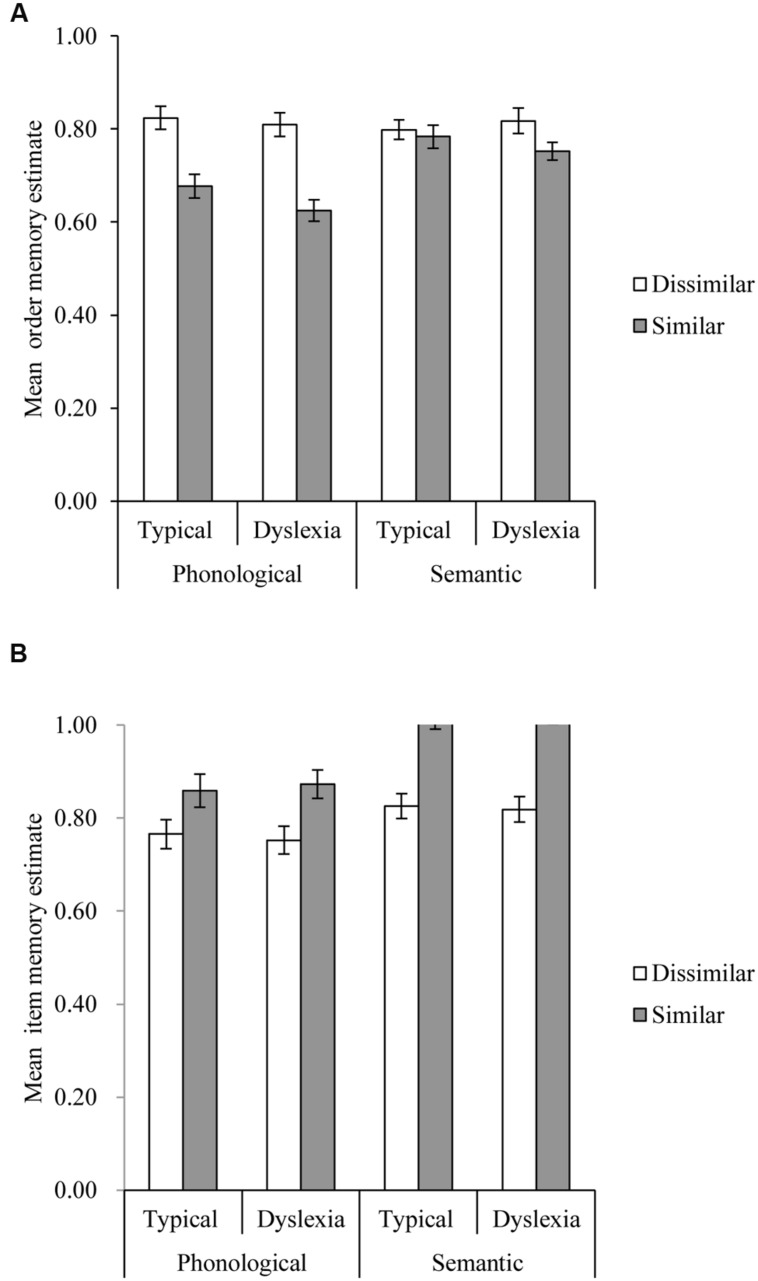
**Order (A) and item (B) estimates in each condition for the self-diagnosed dyslexia and typical groups**.

We then computed the order (Or) and item (I) estimate for each manipulation type and condition separately. The results of these calculations are shown in **Figure 4**. An ANOVA on phonological Or scores with group as a between-subject factor and similarity as a within-subject factor was first conducted. It revealed a significant phonological similarity effect, *F*(1,55) = 69.547, *p* < 0.001, *MSE* = 0.011, η^2^ = 0.281, no significant group effect, *F*(1,55) = 1.334, *p* = 0.253, *MSE* = 0.024, η^2^ = 0.012, and no significant interaction, *F*(1,55) = 0.931, *p* = 0.339, *MSE* = 0.011, η^2^ = 0.004. The corresponding analysis of variance on semantic Or scores showed a significant semantic similarity effect, *F*(1,55) = 6.576, *p* = 0.013, *MSE* = 0.007, η^2^ = 0.025, no significant group effect, *F*(1,55) = 0.042, *p* = 0.838, *MSE* = 0.024, η^2^ = 0.001, and no significant interaction, *F*(1,55) = 2.579, *p* = 0.114, *MSE* = 0.007, η^2^ = 0.010. In both analyses, greater similarity (phonological or semantic) reduced order memory estimates.

The ANOVA of phonological I produced a significant phonological similarity effect, *F*(1,55) = 19.176, *p* < 0.001, *MSE* = 0.017, η^2^ = 0.089, no significant group effect, *F*(1,55) < 0.001, *p* = 0.992, *MSE* = 0.044, η^2^ < 0.001, and no significant interaction, *F*(1,55) = 0.301, *p* = 0.585, *MSE* = 0.017, η^2^ = 0.001. Finally the analysis of semantic I showed a significant semantic similarity effect, *F*(1,55) = 103.774, *p* < 0.001, *MSE* = 0.010, η^2^ = 0.364, no significant group effect, *F*(1,55) = 0.016, *p* = 0.899, *MSE* = 0.024, η^2^ < 0.001, and no significant interaction, *F*(1,55) = 0.362, *p* = 0.550, *MSE* = 0.010, η^2^ = 0.001. In these analyses greater similarity (phonological or semantic) significantly improved item memory estimates.

Finally, in order to explore the associations between reading accuracy and estimates of item or order memory, these variables were correlated with one another and the results of this analysis are illustrated in **Table 2**. Only the correlation between item memory for phonological similar items and non-word reading was marginally significant, *r* = -0.254, *p* = 0.056, *N* = 57, and even this association should be interpreted with a degree of caution given the fact that **Table 2** contains 24 separate correlations.

**Table 2:** Correlations between item and order estimates and reading ability tests.

	Regular words		Irregular words		Non-words	
	*r*	*p*	*r*	*p*	*r*	*p*
Phonological dissimilar Or	–0.015	0.913	0.012	0.932	0.134	0.319
Phonological similar Or	0.026	0.847	0.145	0.281	0.254ˆ*	0.056
Semantic dissimilar Or	0.113	0.403	0.076	0.572	0.117	0.388
Semantic similar Or	–0.07	0.603	–0.074	0.584	0.107	0.426
Phonological dissimilar I	–0.195	0.145	–0.052	0.702	0.113	0.402
Phonological similar I	–0.092	0.494	–0.165	0.219	–0.078	0.563
Semantic dissimilar I	–0.013	0.922	–0.19	0.156	0.142	0.291
Semantic similar I	0.072	0.595	0.108	0.422	0.051	0.707

*^∗^ = *p* < 0.05*.

## Discussion

The present study applied the process dissociation procedure of measuring item and order memory for memoranda with language characteristics manipulated to investigate the verbal short-term memory deficits seen in adults with a self-reported diagnosis of developmental dyslexia. Based on previous studies and the assumption that order memory is independent of language, it was expected that both item and order memory deficits would be present in the self-diagnosed dyslexia group, and that order memory estimates in the phonological and semantically similar trials would be affected by these manipulations to the same amount as that seen in typical readers.

A first point to note is that although the current study adapted the process dissociation paradigm used to distinguish item and order memory by Nairne and Kelley (2004), specifically by presenting more items and allowing immediate rather than delayed recall, the current task produced similar patterns to those seen in this previous work. In particular, phonological similarity between items impaired order memory, a finding consistent with a large body of literature showing that phonological similarity leads to confusions of order (Wickelgren, 1965; Henson et al., 1996). In contrast, both phonological and semantic similarity improved item memory, again in line with Nairne and Kelley (2004) and a number of other studies (Saint-Aubin and Poirier, 1999; Gupta et al., 2005). Our task was therefore sensitive to these experimental manipulations, and would therefore be expected to have the required sensitivity to detect group differences in either item or order memory provided that these effects were of a reasonable size. Indeed, **Figure 1** shows serial position curves for both the inclusion and exclusion tasks that clearly fall below ceiling and above floor, and, as noted above, somewhat greater evidence of an effect of phonological similarity on errors in the exclusion task emerged from our study than was seen by Nairne and Kelley (2004).

In addition, the results of the preliminary reading test showed that the accuracy of reading observed in the individuals with self-reported diagnosis of dyslexia was less than that seen for typical readers for both non-words and irregular words lists, though not significantly impaired for regular words. These key findings confirm that the self-diagnosed dyslexia group assessed here were experiencing statistically significant reading difficulties, and that these were of the form that one would expect in such a sample, even one that self-referred as having a diagnosis of developmental dyslexia. Indeed, the group difference on non-word reading accuracy, where one would most expect individuals with dyslexia to differ from those without dyslexia, was relatively large (an effect size of 0.185). However, contrary to expectations, the present study did not show a general item or order memory deficit in this self-reported diagnosis of dyslexia group. Rather, the two groups performed extremely similarly on the experimental task, leading to comparable item and order estimates (see **Figure 3**). Indeed, the Bayesian analysis of these item and order scores produced positive evidence for the null hypothesis. The evidence for the absence of any group differences on these estimates could have been even greater (for example, posterior probabilities of above 0.95 are considered ‘strong’ evidence). However, it is clear that the current study provides no evidence to support the view that this sample of individuals with a self-reported diagnosis of dyslexia differed from the comparison group on these measures of item and order memory.

However, when the item and order memory estimates were calculated in each condition separately, there was some tentative evidence to suggest that the level of non-word reading was associated with the order memory estimate for phonologically similar materials. Since the reading test showed that non-word reading was significantly impaired among individuals with self-diagnosed dyslexia, one might conclude that adults with dyslexia would exhibit an order memory deficit when the to-be-remembered items place heavy demands on phonological short-term memory in a study with more power to detect such a difference.

Most previous studies have found both item and order memory are impaired in adults or children with dyslexia. This contrast with the present results might be attributed to the fact that non-words or novel words have often been used in those previous item memory or order memory tasks. Another possible reason for the discrepancy with the current findings might be that previous tasks thought to maximize item memory, such as non-word recognition or delayed non-word repetition tasks, depend more on phonological encoding than on short-term memory maintenance processes. Although it have been assumed that dyslexia is associated with inefficient representation of phonological information (Snowling, 1981; Ramus et al., 2003) resulting in an item memory deficit (Burgess and Hitch, 1999; Majerus and D’Argembeau, 2011), this phonological ‘representation’ process might not be constrained by memory processes. If so, then poor performance on tasks that depend on such phonological representation might not necessarily be a sign of poor item memory. Therefore, when item memory is measured using familiar words, as was the case in the current study, any detrimental effect caused by inefficient phonological encoding and subsequent representation might disappear.

Alternatively, although previous studies have shown evidence of problems of serial ordering in dyslexia, it is possible that these reflect a deficit in learning serial order contingencies in the longer-term, more than problems in representing serial order in short-term memory. Szmalec et al. (2011) tested individuals with and without dyslexia on verbal and non-verbal versions of the Hebb repetition paradigm (cf. Hebb, 1961; Couture and Tremblay, 2007). In the Hebb paradigm participants are presented with lists of items for serial recall. The majority of trials are ‘filler’ trials that are different from each other, but a subset of interleaved ‘Hebb’ trials are identical and always present the same items in the same serial order; participants show learning of the ordering of these items across the course of the experiment. Importantly there is evidence to show that while recall performance on filler trials depends on short-term memory capacity, the degree of learning of the ordering of Hebb trials depends on a separate, longer-term learning process (Mosse and Jarrold, 2008). Szmalec et al. (2011) found that individuals with dyslexia were unimpaired, relative to matched controls on recall of filler lists in a Hebb paradigm, but showed impaired longer-term learning of serial order across Hebb trials. Although a more recent replication of this work by Bogaerts et al. (2015) found deficits in both filler trial recall and degree of Hebb learning among a sample of individuals with dyslexia, it remains possible that serial ordering problems associated with the condition are most marked when it comes to learning longer-term contingencies, and are not so apparent in serial order recall tasks that only require the short-term maintenance of the order of a just-presented list.

It should also be acknowledged that the current study relied on self-report of an existing diagnosis of dyslexia, and that formal evidence of this diagnosis was not available for all participants in this group. However, as a group this sample did show significant word reading difficulties, relative to the comparison sample drawn from their peer group, and even more marked difficulties in non-word reading as one would expect from a sample of individuals with a diagnosis of dyslexia. The data from the present study therefore provide some empirical evidence to question the extent to which item and order short-term memory processes are necessarily impaired in individuals with dyslexia. Previous work has suggested that individuals with dyslexia experience item memory deficits resulted from inefficient phonological representation and language-independent order memory deficits. However, the present study found no evidence for the former in our sample and only very limited, suggestive, evidence of the latter. This is potentially consistent with the recent claim (Szmalec et al., 2011) that ordering problems in dyslexia are most apparent in cases when a consistently ordered sequence of items needs to be learnt and maintained in the longer-term, as would be the case in language learning and aspects of reading acquisition.

Of course, one should be cautious when generalizing these conclusions since there are several limitations of the present study. As already noted, although the participants in our ‘dyslexia’ group clearly experienced predicted difficulties in the non-word condition of the reading test, they were a self-referred sample, and, as university students, are not necessarily representative of the broader population with dyslexia. Given that these participants may have been identified as having dyslexia at a younger age, they might have received interventions to improve their reading ability, as well as other abilities that are related to the process of reading, such as short-term memory. The relatively high level of intelligence associated with a university-based sample might also have led participants to circumvent any short-term memory difficulties by long-term learning or through professional training, and these participants may have developed additional memory strategies as a result of their extensive educational experience. Further studies employing children as participants would be need to reduce these effects related to life experience. Second, the mean frequency and familiarity of values of the items used in the phonological and the semantic stimuli sets were significantly different across these sets. Although no reliable interaction between these two effects and the two groups was found in present study, one should bear in mind that individuals with dyslexia might be more sensitive to any change in frequency or familiarity of word stimuli. Further evidence of the interaction between phonological and semantic factors is clearly needed to fully understand any memory difficulties associated with dyslexia. Third, the process dissociation approach adopted here assumes the independence of item and order memory. While there are reasons for making this assumption, for example in the light of the ability of the perturbation model to simulate Nairne and Kelley’s (2004) findings, this independence cannot be guaranteed. Any violation of this assumption would reduce the validity of the process dissociation procedure. In addition, there may be other problems inherent in Nairne and Kelly’s method. Nairne and Kelly’s assumption is that in the exclusion condition, when a participant remembers the identity of an item, but not its order, she or he will never report it. However, this is not necessarily a valid assumption, as participants might be subject to response biases that lead them to use different strategies. For example, some participants might elect to report an item in the exclusion task even when they do not remember its position, neglecting the risk of it being in the exempted position. Other participants might be more conservative and not report any item whose order is forgotten. Furthermore, there may be a degree of overlap between the inclusion and exclusion tasks. For example, in both tasks if one forgets the position of a given item, but remembers the positions of the other five items, the forgotten position can be inferred by simple deduction. Therefore, further evidence for (or against) the Nairne and Kelly method, including tests of the independence of item and order processes in short-term memory and the validity of these tasks, should be sought in the future.

## Author Contributions

XW contributed to the design of the study, the analysis of data, and the writing of the manuscript. YX contributed to the design of the study, data acquisition, the analysis of data, and the drafting of the manuscript. CJ contributed to the design of the study, the analysis plan, and the writing of the manuscript.

## Conflict of Interest Statement

The authors declare that the research was conducted in the absence of any commercial or financial relationships that could be construed as a potential conflict of interest.

## References

[B1] Attout L. Van der Kaa M. A. George M. Majerus S. (2012). Distinguishing verbal short-term memory and language impairment: the importance of short-term memory for serial order. *Aphasiology* 26 355–382. 10.1080/02687038.2011.604303

[B2] Avons S. E. Hanna C. (1995). The memory-span deficit in children with specific reading disability: is speech rate responsible? *Br. J. Dev. Psychol.* 13 303–311. 10.1111/j.2044-835X.1995.tb00681.x

[B3] Baddeley A. Gathercole S. Papagno C. (1998). The phonological loop as a language learning device. *Psychol. Rev.* 105 158–173. 10.1037/0033-295X.105.1.158 9450375

[B4] Bogaerts L. Szmalec A. Hachmann W. M. Page M. P. A. Duyck W. (2015). Linking memory and language: evidence for a serial-order learning impairment in dyslexia. *Res. Dev. Disabil.* 4 106–122. 10.1016/j.ridd.2015.06.012 26164302

[B5] Brady S. Shankweiler D. Mann V. (1983). Speech perception and memory coding in relation to reading ability. *J. Exp. Child Psychol.* 35 345–367. 10.1016/0022-0965(83)90087-5 6842131

[B6] Brock J. Jarrold C. (2004). Language influences on verbal short-term memory performance in Down syndrome: item and order recognition. *J. Speech Lang. Hear Res.* 47 1334–1346. 10.1044/1092-4388(2004/100) 15842014

[B7] Brown G. D. A. Preece T. Hulme C. (2000). Oscillator-based memory for serial order. *Psychol. Rev.* 107 127–181. 10.1037/0033-295X.107.1.127 10687405

[B8] Brown G. D. A. Vousden J. I. McCormack T. Hulme C. (1999). The development of memory for serial order: a temporal-contextual distinctiveness model. *Int. J. Psychol.* 34 389–402. 10.1080/002075999399747

[B9] Burgess N. Hitch G. J. (1992). Toward a network model of the articulatory loop. *J. Mem. Lang.* 31 429–460. 10.1016/0749-596X(92)90022-P

[B10] Burgess N. Hitch G. J. (1999). Memory for serial order: a network model of the phonological loop and its timing. *Psychol. Rev.* 106 551–581. 10.1037/0033-295X.106.3.551

[B11] Coltheart M. (1981). The MRC psycholinguistic database. *Q. J. Exp. Psychol.* 33A, 497–505. 10.1080/14640748108400805

[B12] Coltheart M. Rastle K. Perry C. Langdon R. Ziegler J. (2001). A dual route cascaded model of visual word recognition and reading aloud. *Psychol. Rev.* 108 204–256. 10.1037/0033-295X.108.1.204 11212628

[B13] Couture M. Tremblay S. (2007). Exploring the characteristics of the visuo-spatial Hebb repetition effect. *Mem. Cogn.* 34 1720–1729. 10.3758/BF03195933 17489297

[B14] Critchley M. (1975). “Specific developmental dyslexia,” in *Foundations of Language Development: A Multidisciplinary Approach*, eds Lenneberg E. H. Lenneberg E. (New York, NY: Academic Press), 361–366.

[B15] Crowder R. G. (1979). “Similarity and serial order in memory,” in *The Psychology of Learning and Motivation* Vol. 13 ed. Bower G. H. (New York, NY: Academic Press), 319–353. 10.1016/S0079-7421(08)60086-9

[B16] Estes W. K. (1997). Processes of memory loss, recovery, and distortion. *Psychol. Rev.* 104 148–169. 10.1037/0033-295X.104.1.148 9009883

[B17] Farrell S. Lewandowsky S. (2002). An endogenous distributed model of ordering in serial recall. *Psychon. Bull. Rev.* 9 59–79. 10.3758/BF03196257 12026954

[B18] Gupta P. (2003). Examining the relationship between word learning, nonword repetition, and immediate serial recall in adults. *Q. J. Exp. Psychol.* 56 1213–1236. 10.1080/02724980343000071 12959911

[B19] Gupta P. Lipinski J. Aktunc E. (2005). Reexamining the phonological similarity effect in immediate serial recall: the roles of type of similarity, category cuing, and item recall. *Mem. Cogn.* 33 1001–1006. 10.3758/BF03193208 16496721

[B20] Han L. Kashyap A. Finin T. Mayfield J. Weese J. (2013). “UMBC_EBIQUITY-CORE: semantic textual similarity systems,” in *Proceedings of the Second Joint Conference on Lexical and Computational Semantics*, Atlanta.

[B21] Harm M. W. Seidenberg M. S. (2001). Are there orthographic impairments in phonological dyslexia? *Cogn. Neuropsychol.* 18 71–92. 10.1080/02643290125986 20945207

[B22] Harm M. W. Seidenberg M. S. (2004). Computing the meaning of words in reading: cooperative division of labor between visual and phonological processes. *Psychol. Rev.* 111 662–720. 10.1037/0033-295X.111.3.662 15250780

[B23] Healy A. F. (1974). Separating item from order information in short term memory. *J. Verbal Learn. Verb. Behav.* 13 644–655. 10.1016/S0022-5371(74)80052-6

[B24] Hebb D. (1961). “Distinctive features of learning in the higher animal,” in *Brain Mechanisms and Learning*, ed. Delafresnaye J. F. (Oxford: Blackwell), 37–46.

[B25] Henson R. N. A. (1998). Short-term memory for serial order: the start-end model. *Cogn. Psychol.* 36 73–137. 10.1006/cogp.1998.0685 9721198

[B26] Henson R. N. A. Norris D. G. Page M. P. A. Baddeley A. D. (1996). Unchained memory: error patterns rule out chaining models of immediate serial recall. *Q. J. Exp. Psychol.* 49A, 80–115. 10.1080/713755612

[B27] Jacoby L. L. Yonelinas A. P. Jennings J. (1997). “The relation between conscious and unconscious (automatic) influences: a declaration of independence,” in *Scientific Approaches to the Question of Consciousness*, eds Cohen J. Schooler J. W. (Mahwah, NJ: Erlbaum), 13–47.

[B28] Kramer J. H. Knee K. Delis D. C. (2000). Verbal memory impairments in dyslexia. *Arch. Clin. Neuropsychol.* 15 83–93. 10.1016/S0887-6177(99)00022-0 14590570

[B29] Landauer T. K. Foltz P. W. Laham D. (1998). Introduction to latent semantic analysis. *Discour. Process.* 25 259–284. 10.1080/01638539809545028

[B30] Lee C. L. Estes W. K. (1981). Item and order information in short-term memory: evidence for multilevel perturbation processes. *J. Exp. Psychol. Hum. Learn. Mem.* 7 149–169. 10.1037/0278-7393.7.3.149

[B31] Majerus S. D’Argembeau A. (2011). Verbal short-term memory reflects the organization of long-term memory. Further evidence from short-term memory for emotional words. *J. Mem. Lang.* 64 181–197. 10.1016/j.jml.2010.10.003

[B32] Majerus S. Norris D. Patterson K. (2007). What do patients with semantic dementia remember in verbal short-term memory? Sounds and order but not words. *Cogn. Neuropsychol.* 24 131–151. 10.1080/02643290600989376 18416485

[B33] Majerus S. Poncelet M. Greffe C. Van der Linden M. (2006). Relations between vocabulary development and verbal short-term memory: the relative importance of short-term memory for serial order and item information. *J. Exp. Child Psychol.* 93 95–119. 10.1016/j.jecp.2005.07.005 16154583

[B34] Manis F. R. Seidenberg M. S. Doi L. M. McBride-Chang C. Peterson A. (1996). On the bases of two subtypes of developmental dyslexia. *Cognition* 58 157–195. 10.1016/0010-0277(95)00679-6 8820386

[B35] Martin J. Cole P. Leuwers C. Casalis S. Zorman M. Sprenger-Charolles L. (2010). Reading in French-speaking adults with dyslexia. *Ann. Dyslexia* 60 238–264. 10.1007/s11881-010-0043-8 20872102

[B36] Martinez Perez T. Majerus S. Mahot A. Poncelet M. (2012). Evidence for a specific impairment of serial order short-term memory in dyslexic children. *Dyslexia* 18 94–109. 10.1002/dys.1438 22389071

[B37] Martinez Perez T. Majerus S. Poncelet M. (2013). Impaired short-term memory for order in adults with dyslexia. *Res. Dev. Disabil.* 34 2211–2223. 10.1016/j.ridd.2013.04.005 23644228

[B38] Mason M. (1980). Reading ability and the encoding of item and location information. *J. Exp. Psychol. Hum. Percept. Perform.* 6 89–98. 10.1037//0096-1523.6.1.89 6444996

[B39] Masson M. E. J. (2011). A tutorial on a practical Bayesian alternative to null-hypothesis significance testing. *Behav. Res. Methods* 43 679–690. 10.3758/s13428-010-0049-5 21302025

[B40] Mosse E. K. Jarrold C. (2008). Hebb learning, verbal short-term memory, and the acquisition of phonological forms in children. *Q. J. Exp. Psychol.* 61 505–514. 10.1080/17470210701680779 18300182

[B41] Murdock B. B. (1976). Item and order information in short term serial memory. *J. Exp. Psychol. Gen.* 105 191–216. 10.1037/0096-3445.105.2.191

[B42] Nairne J. S. Kelley M. R. (2004). Separating item and order information through process dissociation. *J. Mem. Lang.* 50 113–133. 10.1016/j.jml.2003.09.005

[B43] Nithart C. Demont E. Majerus S. Leybaert J. Poncelet M. Metz-Lutz M.-N. (2009). Reading disabilities in SLI and dyslexia result from distinct phonological impairments. *Dev. Neuropsychol.* 34 296–311. 10.1080/87565640902801841 19437205

[B44] Pennington B. Van Orden G. Smith S. Green P. Haith M. (1990). Phonological processing skills and deficits in adult dyslexics. *Child Dev.* 61 1753–1778. 10.2307/1130836 2083497

[B45] Plaut D. C. McClelland J. L. Seidenberg M. S. Patterson K. (1996). Understanding normal and impaired word reading: computational principles in quasi-regular domains. *Psychol. Rev.* 103 56–115. 10.1037/0033-295X.103.1.56 8650300

[B46] Plaza M. Cohen H. Chevrie-Muller C. (2002). Oral language deficits in dyslexic children: weaknesses in working memory and verbal planning. *Brain Cogn.* 48 505–512. 10.1006/brcg.2001.1407 12030497

[B47] Raftery A. E. (1995). “Bayesian model selection in social research,” in *Sociological Methodology*, ed. Marsden P. V. (Cambridge: Blackwell), 111–196.

[B48] Ramus F. (2014). Should there really be a “Dyslexia debate?” *Brain* 137 3371–3374. 10.1093/brain/awu295

[B49] Ramus F. Rosen S. Dakin S. Day B. Castellote J. White S. (2003). Theories of developmental dyslexia: insights from a multiple case study of dyslexic adults. *Brain* 126 841–865. 10.1093/brain/awg076 12615643

[B50] Roodenrys S. Stokes J. (2001). Serial recall and non-word repetition in reading disabled children. *Read. Writ.* 14 379–394. 10.1023/A:1011123406884

[B51] Saint-Aubin J. Poirier M. (1999). Semantic similarity and immediate serial recall: is there a detrimental effect on order information? *Q. J. Exp. Psychol.* 52A, 367–394. 10.1080/027249899391115 10428684

[B52] Smith E. Jarrold C. (2014). Demonstrating the effects of phonological similarity and frequency on item and order memory in Down syndrome using process dissociation. *J. Exp. Child Psychol.* 128 69–87. 10.1016/j.jecp.2014.07.002 25089885

[B53] Snowling M. (1981). Phonemic deficits in developmental dyslexia. *Psychol. Res.* 43 219–234. 10.1007/BF00309831 7302091

[B54] Snowling M. Goulandris N. Defty N. (1996). A longitudinal study of reading development in dyslexic children. *J. Educ. Psychol.* 88 653–669. 10.1037/0022-0663.88.4.653

[B55] Szmalec A. Loncke M. Page M. P. A. Duyck W. (2011). Order or disorder? Impaired Hebb learning in dyslexia. *J. Exp. Psychol. Learn. Mem. Cogn.* 37 1270–1279. 10.1037/a0023820 21604915

[B56] Tijms J. (2004). Verbal memory and phonological processing in dyslexia. *J. Res. Read.* 27 300–310. 10.1111/j.1467-9817.2004.00233.x

[B57] Tree J. J. Kay J. (2006). Phonological dyslexia and phonological impairment: an exception to the rule? *Neuropsychologia* 44 2861–2873. 10.1016/j.neuropsychologia.2006.06.006 16879843

[B58] Wickelgren W. A. (1965). Short-term memory for phonemically similar lists. *Am. J. Psychol.* 78 567–574. 10.2307/1420917 5839924

